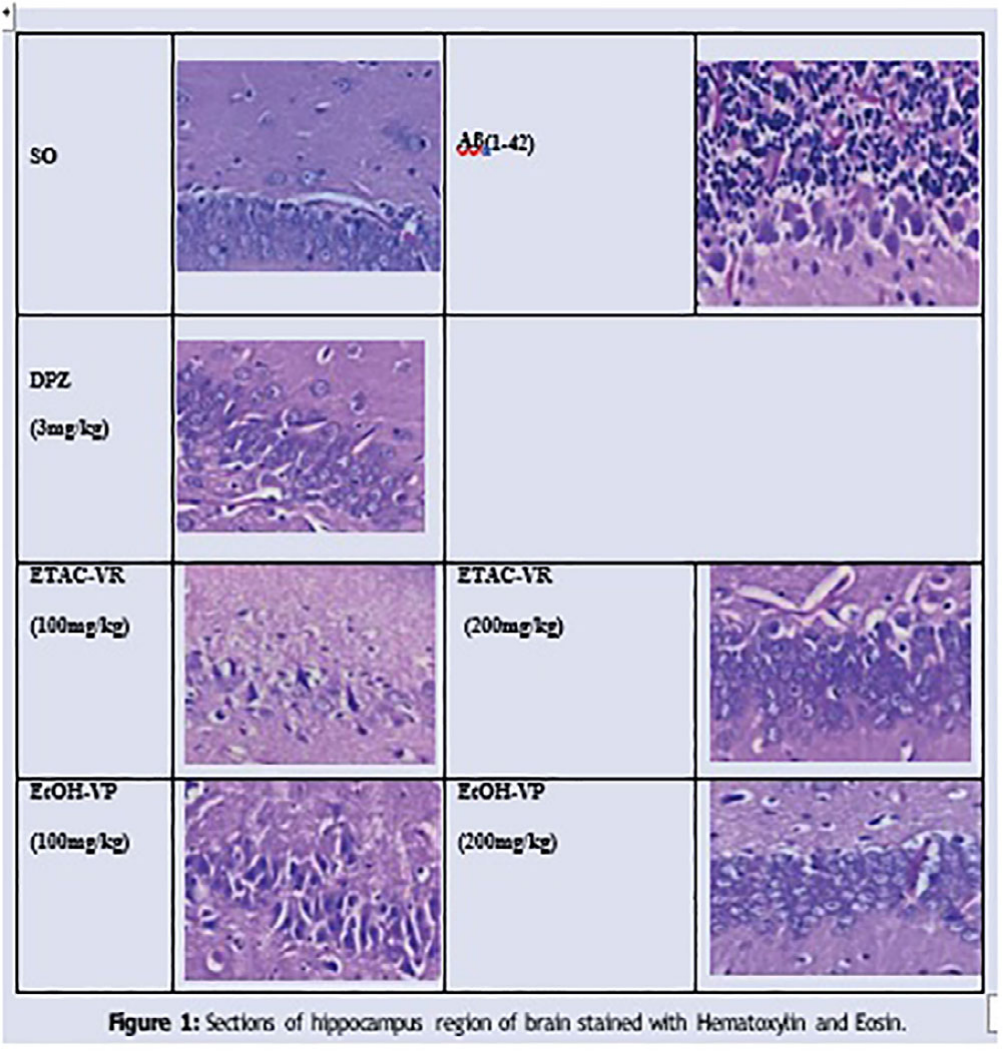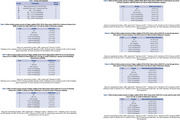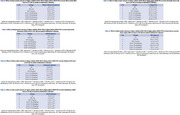# Anti‐Alzheimer Effects of Vigna Radiata and Vigna Pilosa in Rats Induced with Beta Amyloid

**DOI:** 10.1002/alz70856_097099

**Published:** 2025-12-24

**Authors:** Ankur Joshi, Dr Neelesh Malviya, Dr Sapna Malviya, Priyanka Soni, Purva Khemani, Dr Neetesh Kumar Jain, Dr Anil Kharia

**Affiliations:** ^1^ MANDSAUR UNIVERSITY, MANDSAUR, India; ^2^ Modern Institute of Pharmaceutical Sciences Indore, Indore, India; ^3^ Smriti College of Pharmaceutical Education, Indore, M.P., India, Indore, India; ^4^ Modern Institute of Pharmaceutical Sciences, Indore, Indore, India; ^5^ SRI AURVINDO INSTITUTE OF PHARMACY, INDORE, India

## Abstract

**Background:**

Alzheimer's disease is the commonest kind of dementia. Both intracellular neurofibrillary tangles (NFTs) and extracellular senile plaques, also known as amyloid plaques, are characteristic features of the condition. Both the Vigna radiata and the Vigna pilosa are plants that are used in numerous Ayurvedic formulations that are utilized in the treatment of dementia and ailments that are associated to it.

**Method:**

Purpose of assessing the neuroprotective impact of these plants on an amyloid‐β (Aβ) 1‐42 model of Alzheimer's disease in rats, the current study was conducted. This study lasted for a total of twenty‐one days. After the recovery time following the injection of Aβ1‐42 intravenous, beginning on the eighth day, the ethyl acetate extract of Vigna radiata and the ethanolic extract of Vigna pilosa were administered at doses of 200mg/kg and 400mg/kg, respectively. Additionally, the dose of Donepezil was administered at a dose of 3mg/kg once day until the 21st day. Both the radial maze test and the Step‐through Passive Avoidance Test were utilized in the cognitive behavioural research that was carried out. Euthanasia was performed on the animals, and the brains were removed and analyzed for antioxidant parameters and levels of brain cytokines. The animals were then returned to their owners. It was determined by histological analysis that the brain tissues were examined.

**Result:**

In both the radial arm maze task and the step through passive avoidance test, the rats' cognitive capabilities were greatly improved as a result of the treatment with the extracts. Additionally, it decreased oxidative stress, which was demonstrated by the decreased levels of lipid peroxide and nitric oxide, in addition to the increased levels of antioxidant enzymes such as catalase, superoxide dismutase, and reduced glutathione. A dose‐dependent reduction in the concentration of neuroinflammatory markers was achieved by the treatment, which resulted in a reduction in the severity of neuroinflammation in rats.

**Conclusion:**

The findings allow for the conclusion that the plants Vigna radiata and Vigna pilosa have favourable effects in the improvement of cognitive impairment in Alzheimer's disease (AD). These benefits are achieved through the reduction of oxidative stress and neuroinflammation. Amyloid‐β, Vigna radiata, and Vigna pilosa